# Assessment of Patients with von Willebrand Disease with ISTH/BAT and PBQ Scores

**DOI:** 10.4274/tjh.galenos.2019.2019.0446

**Published:** 2020-02-20

**Authors:** Fatma Burcu Belen Apak, Elif Gülsüm Ümit, Yağmur Zengin, Melike Sezgin Evim, Ekrem Ünal, Hasan Mücahit Özbaş, Can Acıpayam

**Affiliations:** 1 Başkent University Faculty of Medicine, Department of Pediatric Hematology Oncology, Ankara, Turkey; 2 Trakya University Faculty of Medicine, Department of Hematology, Edirne, Turkey; 3 Başkent University Faculty of Medicine, Department of Biostatistics, Ankara, Turkey; 4 Uludağ University Faculty of Medicine, Department of Pediatric Hematology Oncology, Bursa, Turkey; 5 Erciyes University Faculty of Medicine, Department of Pediatric Hematology Oncology, Kayseri, Turkey; 6 Giresun University Faculty of Medicine, Department of Hematology, Giresun, Turkey; 7 Kahramanmaraş Sütçü İmam University Faculty of Medicine, Department of Pediatric Hematology and Oncology, Kahramanmaraş, Turkey

**Keywords:** Von Willebrand Disease, Pediatric Bleeding Questionnaire, ISTH/BAT score

## To the Editor,

The Turkish Society of Hematology initiated the Turkish Hemophilia Masterclass Academy program in 2016 to encourage young hematologists entering the field of hemophilia. The program involved 6 months of training, supported by monthly exams. We, as a group of mentees from the Hemophilia Masterclass Academy, aimed to evaluate the bleeding phenotype of patients with von Willebrand Disease (VWD) using the International Society of Thrombosis and Haemostasis-Bleeding Assessment Tool (ISTH-BAT) and the Pediatric Bleeding Questionnaire (PBQ) scores and investigate the correlation of von Willebrand factor antigen (VWF:Ag) levels and bleeding scores of the patients, as well as present the initial output of our Masterclass Academy.

The study included 62 patients (aged 3-61 years) with the diagnosis of VWD (54 patients with VWD type 1 and 8 with VWD type 3). The ISTH-BAT and PBQ were administered to patients who were ≥18 years old and <18 years old, respectively. Informed consent was obtained from all patients.

The VWF:Ag levels, ristocetin cofactor activity (VWF:Ricof), and FVIII levels were retrospectively reviewed from the patient records. The cut-off point for a positive score was accepted as ≥2 for the PBQ and ≥3 for the ISTH-BAT. Statistical analysis was performed using SPSS 17.0. Demographic findings, median bleeding scores, and VWF levels are presented in Table 1. Epistaxis, superficial bleedings, bleeding from minor wounds, oral bleeding, umbilical bleeding, and muscle hematoma were found to be statistically significant in showing dependence between the diagnostic status and the bleeding symptoms (p<0.05). The study aimed to investigate the correlation between the VWF:Ag levels and the bleeding scores in our patients. Our study showed that VWF:Ag levels were inversely correlated with the bleeding scores of the patients (Table 2).

The evaluation of bleeding scores dates back to the Vicenza score that was used in VWD patients [1,2,3]. In 2009, Bowman et al. [4] published the PBQ, and it was investigated in our population with VWD previously [5]. In 2010, the ISTH-BAT score was established by the ISTH/SSC Joint Working Group [6,7]. One of the limitations of our study was the use of the PBQ in children and ISTH-BAT in adults, which might have caused heterogeneity in the evaluation of the scores. Moreover, the lack of a control group, consisting of patients who had bleeding symptoms but were normal by hemostatic tests, left us unable to compare the VWD patients and normal individuals. Although the median total scores of the types 1 and 3 VWD groups were compatible with previous studies, the positive scores reported in each subgroup for epistaxis, oral cavity bleeding, cutaneous bleeding, and bleeding after minor wounds were found to be lower compared to those in the previous literature [4,5,8]. This may be explained by the inclusion of “low VWF levels” (intermediate levels of VWF:Ag, 30-50 IU/dL) in the group with type 1 VWD. People with “low VWF levels” falsely labeled as “VWD type 1 patients” may have lower reported bleeding scores compared to true VWD patients, leading to low positive scores. Our study shows that the ISTH/BAT and PBQ can be useful in the evaluation of the bleeding symptoms of patients. Further studies with larger patient and control groups are warranted to show the usage of bleeding scores in daily outpatient practice. We, as the mentees of the Hemophilia Masterclass, feel much appreciation to our mentors and the Turkish Society of Hematology for their contributions to our progress in the field of hemophilia.

## Figures and Tables

**Table 1:**
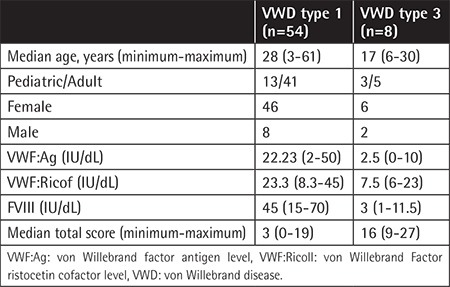
Characteristics of von Willebrand type 1 and type 3 patients.

**Table 2:**
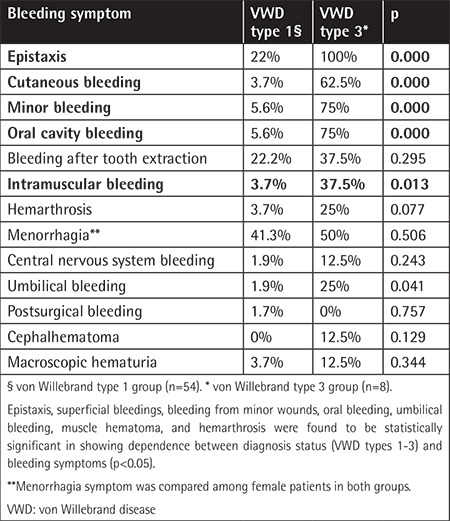
Bleeding scores positivity regarding each symptom in von Willebrand type 1 and type 3 patients.
